# Review of techniques and models used in optical chemical structure recognition in images and scanned documents

**DOI:** 10.1186/s13321-022-00642-3

**Published:** 2022-09-09

**Authors:** Fidan Musazade, Narmin Jamalova, Jamaladdin Hasanov

**Affiliations:** 1 School of Engineering and Applied Science, The George Washington University, Washington, DC United States; 2 School of IT and Engineering, ADA University, Baku, Azerbaijan

**Keywords:** InChI, Chemical images, Molecules, Computer vision, Transformers, ViT, LSTM, Attention, Augmentation

## Abstract

Extraction of chemical formulas from images was not in the top priority of Computer Vision tasks for a while. The complexity both on the input and prediction sides has made this task challenging for the conventional Artificial Intelligence and Machine Learning problems. A binary input image which might seem trivial for convolutional analysis was not easy to classify, since the provided sample was not representative of the given molecule: to describe the same formula, a variety of graphical representations which do not resemble each other can be used. Considering the variety of molecules, the problem shifted from classification to that of formula generation, which makes Natural Language Processing (NLP) a good candidate for an effective solution. This paper describes the evolution of approaches from rule-based structure analyses to complex statistical models, and compares the efficiency of models and methodologies used in the recent years. Although the latest achievements deliver ideal results on particular datasets, the authors mention possible problems for various scenarios and provide suggestions for further development.

## Introduction

The analysis of printed or digitized chemical structures is an important task in education, research and development of chemical products. Molecular structures contained in conventional scientific publications, documents and textbooks usually come in the form of images and annotated text. Structural formulas are represented as chemical graphs, where vertices are molecules and edges represent bonds among them. This data, especially that from older papers, is not digitized (not only as images, but also in a descriptive language), so extracting useful information involves a lot of manual effort. Moreover, there is no standardized and centralized database for the storage and retrieval of chemical structure information. Automatic recognition of images containing such structures and their conversion to standard chemical identifiers is required to increase the efficiency of scientific processes, reduce manual efforts and help capture the data in a standard way for subsequent data mining opportunities. Optical Chemical Structure Recognition (OCSR) addresses the problem of the translation of a chemical structure provided as a graphic representation into the corresponding chemical formula.

The problem of chemical structure recognition can be seen as a mixture of image processing, classification and sequence modeling. The source of the digital information, which is inputted to the recognition system, plays an important role in designing the recognition model: the scanned documents usually contain more noise and information loss compared to the images generated by software tools or written by a digital stylus. The scanned images are also categorized as printed and handwritten. Due to freedom in writing styles of authors,the handwritten structures bring complexities during the scanned or offline document analysis. For the real-time or online recognition systems, the stokes play an important role, such that the vector graphics is additionally paired with the temporal information. Since the problem that OCSR deals with the existing printed material, the research is this area prevails the online chemical structure analysis.

Rule or heuristics-based systems apply an expert’s reading approach to construct a meta-descriptor that contains the strokes, shapes, letters and their connections retrieved from a graphic image. The final formula is generated based on the rule applied to the data of the meta-descriptor. From the Machine Learning (ML) perspective, the identification of an image with a unique text string can be considered as a classification problem, but the possibility of having an unlimited number of classes changes the course of solution to the image captioning problem.

The development of the first OCSR systems started in the early 1990s [1–5]. Due to limitations in computational resources and the early stage of Computer Vision oriented Machine Learning algorithms at that time, those solutions mainly used more rule-oriented heuristics, supported by the classic Artificial Intelligence and optical character recognition algorithms. The image analysis was mainly done with the classic image processing algorithms like morphological operations, skeletonization and template matching. By this time, with the developments in hardware, cloud computing and Deep Neural Networks (DNN), all the OCSR stages started to move completely into Machine Learning models, and delivered exceptional results. Employment of attention-based and context-aware image classification models [6–10] removed the necessity of having separate pre-processing phases like noise removal of image restoration. Modern NLP models [11–15] that are capable of being trained to understand and generate complex-structured sequences replaced the expert-driven rules on molecular structure, bonding and formatting details. Despite their approximation and learning power, as all statistical learning machines, ML-based models depend on representative training samples, and are therefore sensitive to variations; different structural representations of the same formula visually different from the training sample may not be recognized successfully. Similar shortcomings of ML-based solutions motivate researchers not to abandon the rule-based approach completely [16–19].

This review paper primarily focuses on the characteristic problems of the OCSR and analyzes the previous approaches without outlining any of them in terms of performance or accuracy. Conclusions made after each review section show the opportunities related to and shortcomings of the particular approach. The following chapter introduces the main chemical structure identifiers and briefly explains their specifics. The various OCSR solutions, categorized as rule-based and Machine Learning-based are listed in chapter “Approaches, methods and models”. This chapter goes through the evolution of the OCSR development by each approach and provides an analysis of their strong points and shortcomings. Additional attention has been paid to solutions that delivered progressive results throughout their evolution and datasets used by various models. The evaluation criterion which is not yet standardized for scoring the performance of OCSR is also brought to the discussion. The review work provided by the authors is summarized with the conclusions and recommendations for the OCSR developers. The summary mentions the successful strategies for both rule and ML-based systems and shares ideas on possible improvements.

### Chemical structure identifiers

The chemical formulas are currently encoded by standard chemical identifiers that contain unique textual and numeric sequences. There are a number of such identifiers in the industry, including InChI (International Chemical Identifier), UNII (Unique Ingredient Identifier), CAS RN (Chemical Abstract Service Registry Number) among others. These identifiers encode chemical structures in a standardized way, hence easing their search in databases and on the web.

SMILES (Simplified Molecular-Input Line-Entry System) is a way to represent molecular structures by using short American Standard Code for Information Interchange (ASCII) strings. This identifier builds a connection graph, i.e. vertices and edges, of a molecular structure. This is usually considered an easier and more intuitive approach to compactly represent molecular structures compared to alternatives. For instance, below is the SMILES identifier for ethanol:

$$\begin{aligned} \hbox {CCO}. \end{aligned}$$

However, one of the major disadvantages of using SMILES is that one chemical structure can have more than one SMILES representation, depending on the algorithm in use. Various commercial and scientific software tools were historically developed in separate ways and hence differ in how the final SMILES string is produced [20].

Various alternatives have been suggested for the improvement of the SMILES representation or to address the main shortcomings of it. SMILES Arbitrary Target Specification (SMARTS) [21] is a language designed for the specification of substructures using rules that are extensions of SMILES. Such description helps matching the molecules with a particular substructure in a database. [22] provides a detailed description of the specifications, implementation details and applications of the SMILES and SMARTS. DeepSMILES [23] is a machine learning oriented syntax model that is intended for the improvement of the predecessor’s syntactical problems. The use of close parentheses avoids the problem of unbalanced parentheses, where the number of parentheses define the branch length. Additionally DeepSMILES uses only a single symbol indicating the ring size at the ring closing location, which avoids the problem of pairing ring closure symbols. DeepSMILES mainly solves the most syntactical issues encountered in generate graphs, but does not deal with semantic constraints that are introduced by the specific domain. SELF-referencIng Embedded Strings (SELFIES) [24] is a string-based representation of molecules which is able to describe any molecule and guarantee its validity. SELFIES is targeted as an input for generative machine learning models, which does not require the adaptation of the learning model to the structure.

InChI is a categorically different approach that mainly focuses on the uniqueness of the definition rather than readability. The InChI identifier that delivers one unique string per chemical structure is generated by the following process:

1. Normalization, which is the process of removing useless information from the structure, such as unneeded atoms and/or bonds;
2. Canonicalization, which is the process of creating a unique number label for each atom;
3. Serialization, which is the process of generating a string of textual and numeric characters.

The final InChI string is a layered representation of a chemical structure, in a sense that the string is composed of parts (layers), each of which carries its own purpose (e.g., charge layer, isotopic layer, etc.). For example, below is the InChI label for methanol:

$$\begin{aligned} \hbox {InChI = 1S/CH40/c1-2/h2H,1H3,} \end{aligned}$$

where CH40 is the elemental composition layer, c1-2 is the connection layer and h2H, 1H3 is the hydrogen count layer [25].

To note, InChI does not come without its own set of issues. In particular, here is a list of some of its ongoing challenges [26]:

1. Not all chemical structures are yet accounted for, especially when it comes to more complex formulas.
2. InChI is not yet implemented properly for some stereochemistry types.
3. InChI is not as human-readable as SMILES.

A well-defined chemical structure recognition system is of importance due to its applicability in such areas as medical research in commercial and non-commercial (e.g., educational) settings. For example, there are currently such chemistry databases available, including ChemSpider (http://www.chemspider.com/) and PubChem (https://pubchem.ncbi.nlm.nih.gov/) that facilitate search by chemical structures using InChI. Automatic recognition and labeling of molecular structure images, and in particular those contained in older publications, is hence a requirement for expanding the existing chemical knowledge base and creating innovative solutions to increase the efficiency of scientific work.

### Approaches, methods and models

The image captioning is a generation of the output and unconstrained in length, which depending on the tasks, synthesizes the given image’s description or characteristics. The image captioning is achieved by various techniques and models, like structure analysis, rule-based models, recurrent networks, attention models and Natural Language Processing. Overall, the approaches on OCSR problems can be categorized as *Rule-based* and *Machine Learning*-based systems. The following part of the chapter describes each of the approaches and previous works where they have been applied.

#### Rule-based systems

Rule-based systems try to imitate the perception model of a human, which in OCSR is the detection of characters and shapes, understanding the connection of the lines and constructing a formula based on the given analysis. Most rule-based approaches follow the pattern of recognizing atoms and bond lines, image vectorization and reconstruction of connection tables or graphs [3, 27–29]. A number of studies focus on detecting hexagonal and pentagonal structures based on the predefined rules [30]. Circle detection was also performed as a separate step [31]. Optical recognition is largely based on a set of rules embedded into the backbone of the system.

Some early methods, like Optical Recognition of Chemical Graphics (OROCS) and Chemical Literature Data Extraction (CLiDE) focused on using polygon-like shaped boxes to separate the parts of image as a part of image preprocessing [4, 5, 32]. These methods employed Optical Character Recognition (OCR) to identify the characters in the image with the purpose of constructing labels for the molecule. Despite being able to solve the task of chemical image labeling, these tools were commercial and were not used by the research community [33].

The first publicly available tool in this domain was Optical Structure Recognition Application (OSRA) [34], which was published in 2009. The techniques used in OSRA were highly similar to the previous tools, and involved predefined preprocessing steps as well as OCR tools to identify atom labels. Different from its predecessors, OSRA used two OCR systems instead of one and analyzed images in three different resolutions to keep the best result according to the confidence estimation function. Authors of [34] also raise important questions on the evaluation criteria, which still remain relevant. As in all early OSCR implementations, OSRA also operates with a limited dataset, which included only 66 images of various resolutions and color depths. The calculation of the successful recognition rate in OSRA is specific and make it hard to compare the model to the others. Using Tanimoto distance as a gauge, the authors consider 4 separate levels of the performance evaluation. Imago [35], another open-source system published after OSRA had a similar approach with slightly different implementation details. Although the paper describes the implementation procedures in detail, the performance of the algorithm is described more qualitatively, rather than quantitatively.

An alternative approach was used by the authors of Markov Logic OCSR [36], which utilized probabilistic inference over logical worlds. The advantage of this method is evident when working with low-quality databases given their non-deterministic characteristics. However, the system exposes shortcomings with adjacent or broken characters, repeating units and cases when characters touch the shapes. Other tools are also present that implement different techniques, such as chemoCR [37], which combines pattern recognition techniques with supervised machine learning concepts.

Another rule-based approach that used Support Vector Machines (SVMs) was published by Stanford University [38], aiming to recognize hand-written chemical structures with the following steps:

- Text recognition using scale-invariant template matching. There were 6 templates in total, including ”O”, ”H”, ”OR”, ”RO”, ”N”, and ”OH”. A Gaussian filter was used in combination with the spatial pyramid sliding. The output of this step was used in the next steps.
- The image is cleaned from the text using the results of the previous step.
- Bond and corner detection. A broad Harris corner detector was used as the basis of a corner detection algorithm. A Douglas-Peucker algorithm was used to identify D-points, while the Harris algorithm focused on C- and T-points.
- Bond detection. Hough transform was used to only detect bonds. The use of Hough transform was previously concentrated not only on detection but also on classification of bonds.
- Bond classification. A multi-class logistic regression classifier, a linear SVM, and a decision tree were used to classify the bonds detected previously.
- Association of corners to atoms and the groups.

**Fig. 1:**
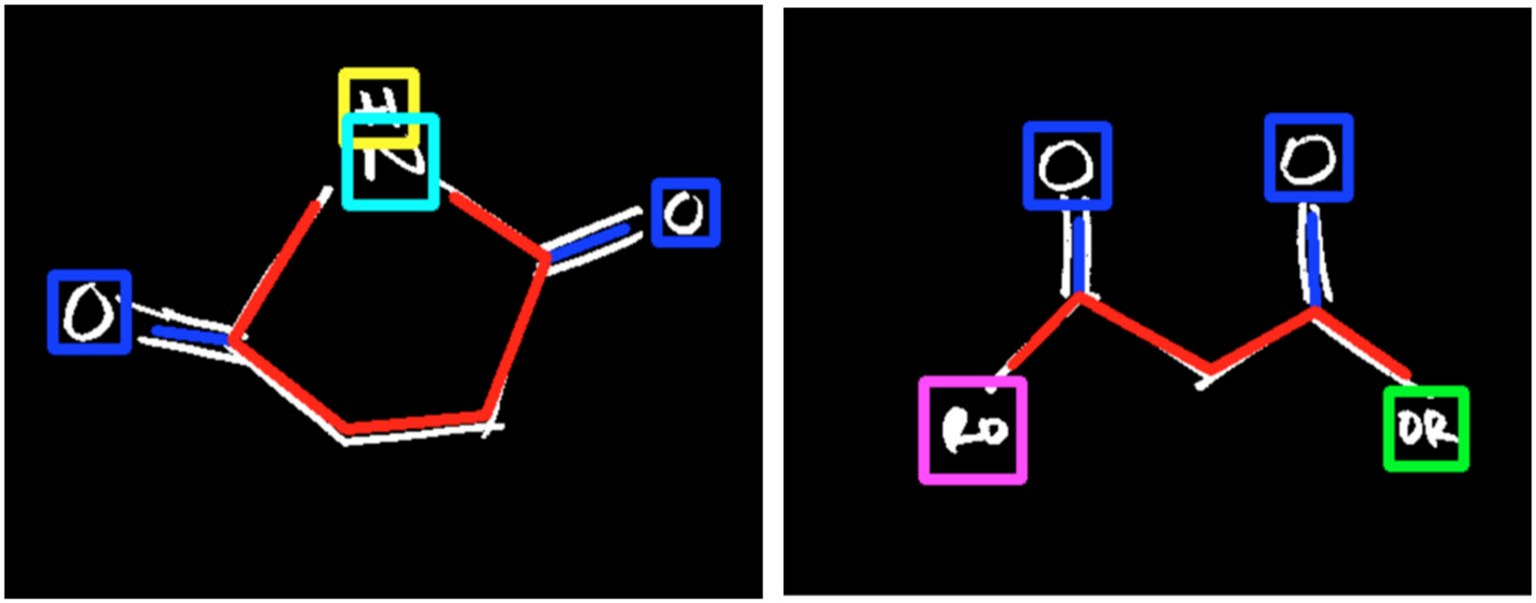
Two correctly classified examples—Stanford study [38]

The study used only 360 images to train and test the algorithm. Out of those 360 images, only 94 were classified correctly. The correct classification examples are shown in Fig. 1. Even though the performance on the selected dataset is low, the authors claim that it performs well on the challenging areas of the previous algorithms.

**Fig. 2:**
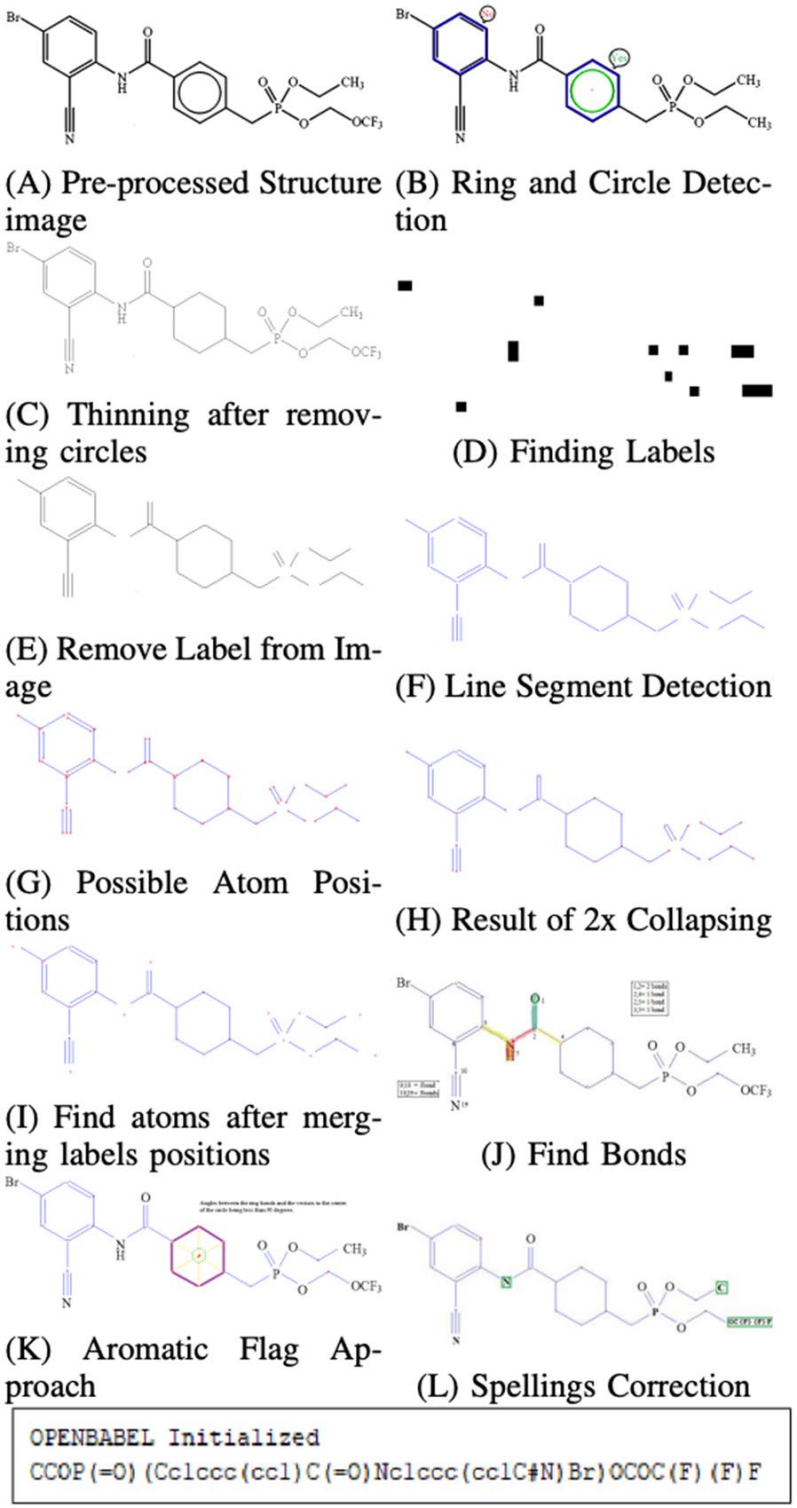
CSR recognition process [18]

[18] published in 2019 describes an improved 2D chemical structure recognition system targeted for OCSR. The pipeline starts with image pre-processing steps, such as blurring and thresholding and applies length smearing and text-region filter. The molecule image extraction from the document if followed by the recognition process. The first step in the recognition process involves ring detection, thinning, finding labels using OCR, and removing those labels from the skeletonized image. Atoms and bonds are discovered using labels and line information retrieved using the line segment detector algorithm. Finally, Open Babel pre-processing is done, which is followed by the assembly of the final structure. The process is illustrated in Fig. 2. Among 50 images used for the test, the success rate was 86%, which is 12% more than that of OSRA.

In [19] authors propose a component-detection-based approach for interpreting the spatial structure of off-line handwritten chemical cyclic compound structure formulas. The work combines the technique with deep learning and proposes the Non-Maximum Area Suppression algorithm to improve the detection results. The experiments on 2100 self-collected dataset achieved 89.6%.

**Table 1:** Performance of the rule-based approaches

OCSR system	Year	Dataset	Evaluation	Results (%)
OSRA	2009	66 images, 215 structures	Tanimoto	95
chemoCR	2011	1000 structures	Structure match	65.6
Markov Logic OCSR	2014	937 images—subset of produced by the USPOCW	InChI match	79.1
			Tanimoto	92.9
		869 images from ChemInfty	InChI match	35.1
			Tanimoto	77.6
OCSR in [38]	2016	360 structures	Structure match	26.1
OCSR in [18]	2019	50 images	Structure match	86
OCSR in [19]	2021	2100 images	Structure match	89.5

Table 1 lists the performance of the mentioned rule-based OCSR systems. Evaluation of the rule-based systems and their comparative analysis is a challenging task due to the following reasons:

1. At early times of rule-based system development, there were no standardized or widely available datasets. Almost each system refers to a dataset either created by itself or selected from various available sets.
2. The sizes of samples in datasets are low compared to those used in modern ML-based OCSRs.
3. Unlike ML-based approaches that require training, validation and test datasets, rule-based models do not require such separation. This approach makes the overall performance questionable - have the rules been developed based on the dataset samples or just by the expert’s procedures? A model that was fine tuned to cover the majority of the samples, cannot be considered as high-performing, since the fair evaluation definitely needs to be done on a completely new dataset.
4. Evaluation criteria was mentioned briefly in the papers, without any implementation details. The same structure and string comparison could be implemented in various ways, which might affect the accuracy of the system positively.

In their experimental results some of the papers show different accuracy for the previously created models. For instance, the results of OSRA in later works is mentioned with a lower score than declared in the original paper. The authors of this paper do not intend to re-gauge the performance of the mentioned models, since such an experiment would be technically impossible to set.

#### Gaps of rule-based systems

The following is a list of pain points encountered in many of the rule-based systems:

- There are too many rules in chemistry and it is quite common that sets of rules embedded into the systems are not comprehensive.
- Rule-based systems typically perform worse on images with complex features, ambiguities and presence of a noise.
- These systems are limited to the given rules—the representations that are not considered by chemical experts might not be recognized at all.
- These systems are noise sensitive—removal of a joint, spot or discontinuity in lines may mislead the system.

#### ML-based systems

Different statistical learning methods have been tested for OCSR problems. Some implementations based on Kohonen networks and SVMs were used to segment images into their constituent objects, such as chemical graph elements and textual symbols [37, 39]. Convolutional Neural Network (CNN)-related solutions have also been presented and achieved significant results [40, 41]. Due to the simplicity of their design and generalization power, ML-based systems have been gaining popularity in the last two decades. This chapter provides an overview of recent ML-based methods.

##### Recognition of handwritten chemical organic ring structure symbols using CNNs

**Fig. 3:**
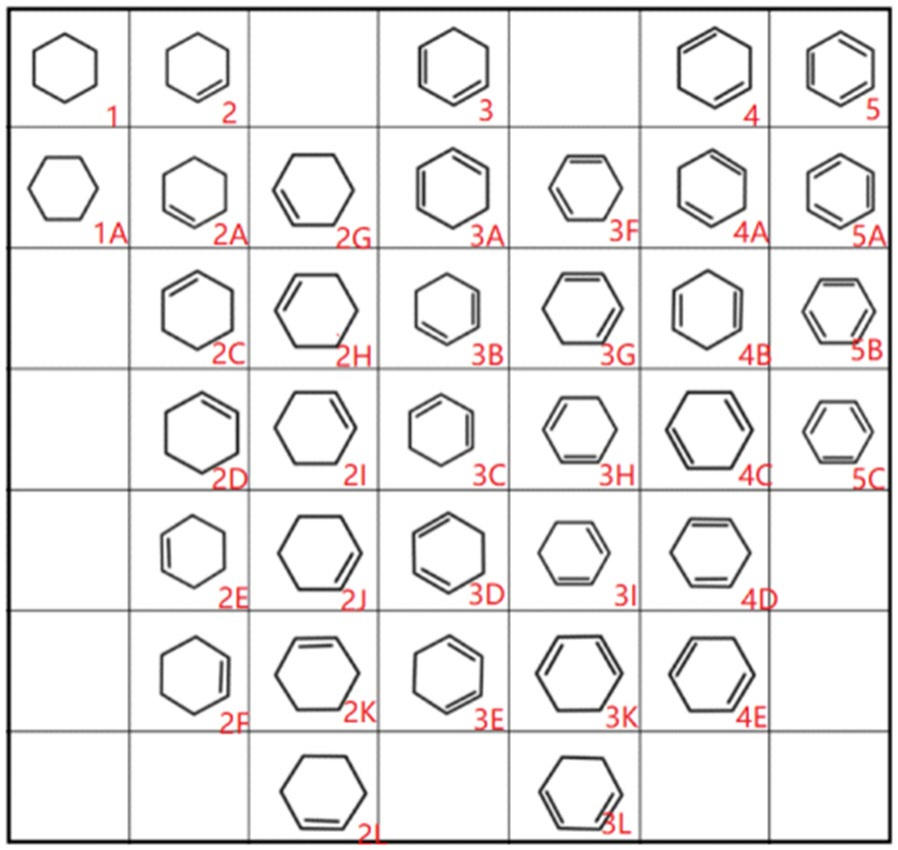
Organic ring structures [40]

This approach is focused on the recognition of ring structures from handwritten images [40]. The method uses transfer learning based on 16 and 19-layered deep convolutional neural networks, VGGNet-16 and VGGNet-19 respectively. The architecture consists of convolutional kernels of size $3 \times 3$, and maximum pooling layers of $2 \times 2$. In this study, 5 standard ring structures are used to derive the total of 36 structures (Fig. 3). The dataset of images drawn by 200 people with augmentation contains 3600 images. 36-class recognition accuracy for VGGNet-19 was 80%, while 5-class alternative achieved more than 92% accuracy. The authors suggest that increasing the dataset size might help improve the performance of the 36-class version of the model.

##### Molecular structure extraction from documents using deep learning (MSE-DUDL)

This approach, developed by Staker et al. [42], consists of two parts:

- U-Net based segmentation model to detect the chemical structure, and
- Structure prediction using attention and Grid Long Short-Term Memory (Grid LSTM).

The label generation starts with the preprocessing, which includes image transformations like binarization and scaling. Segmentation is performed at the full resolution level whereby several masks at different resolutions get averaged to end up with better results (i.e., high confidence pixels form masks at the same resolution as the original input images). The images are first downsampled and then upsampled along the network’s workflow to ensure that the image resolution remains the same. Next, the extracted images are passed on to another deep neural network that follows the encoder-decoder strategy to predict SMILES strings. First, the inputs are encoded into a space vector using a CNN and then they are decoded back into a character sequence (one character at a time) using an RNN (Recurrent Neural Network). In particular, Grid LSTM is used to predict characters based on previous cell states, current attention and previous outputs [33]. The training was performed using the PubChem, Indigo and United States Patent and Trademark Office (USPTO) datasets (achieving a validation accuracy in the range of 77–82%), and the model was tested on Valko (accuracy of 41%) and proprietary datasets (accuracy of 83%). Disadvantages of this model include the following:

- Super-atoms were not well-represented in the datasets used and generally, there was an insufficient sampling of various atoms.
- The datasets used did not encompass all the possible chemical structures and hence, some of the more complex features were not tested for.
- The model was tested on low resolution images only.
- Chemical macrocycles with large rings were not well predicted (which could also be due to the imbalance in the dataset).
- Inverted images (i.e., white structures on a black background) were not well recognized.
- Specifically, the model performed worse on images with too much downscaling, many structures present at the same time and inverse structures.

##### Deep learning for chemical image recognition (DECIMER)

The idea behind DECIMER [43] is to use show-and-tell neural networks, initially developed as an image annotation system targeted to chemical structure recognition: the system accepts an image as an input and produces a SMILES encoding. The dataset was created using the Chemistry Development Kit (CDK), which could potentially produce an unlimited amount of training data. The input to CDK was PubChem data, which was also used in other ML-based systems. The input images did not receive any manipulations, except for one random rotation per molecule. The model that was designed as an autoencoder-based network consists of two parts [43]:

1. Encoder network: CNN with a single fully connected layer and Rectified Linear Unit (ReLU) activation function.
2. Decoder network: RNN, consisting of Gated Recurrent Unit (GRU) and two fully connected layers.

Different SMILES versions are used for prediction, with DeepSMILES being the most accurate one to predict. The overall workflow can be summarized as follows:

- Images are converted into feature vectors using Inception V3.
- In parallel, DeepSMILES are tokenized via a tokenizer and the unique tokens are stored.
- Image vectors are fed to the encoder, while tokens are fed to the decoder networks. The training process uses Adam optimizer and Sparse Categorical Cross Entropy as a loss function.

Training process took around 27 days on the NVIDIA Graphics Processing Unit (GPU). The output was being evaluated based on the Tanimoto similarity score. A score of 0.53 was reached with DeepSMILES. The authors claim that increasing the amount of data to 50–100 million could improve the performance significantly, but that would require several months of training on a single GPU.

##### DECIMER segmentation

Another version of DECIMER, called DECIMER Segmentation was introduced in 2021 [44]. The flow of the new model consists of two primary stages:

1. Detection: a deep neural network generates masks to define positions of chemical elements in the document. The annotation of images was done with the use of the Visual Geometry Group (VGG) annotator. Masks are applied to indicate whether or not the pixel belongs to a chemical structure.
2. Expansion: mask is expanded to completely cover the image. The procedure involves image binarization and binary dilation. With the use of mask expansion, the proportion of completely segmented structures rose to 99.8%.

The approach is claimed to work with only bitmap images as opposed to vector images in PDF, which are more common in modern articles. Overall, 91.3% of chemical structures were detected by the model.

##### DECIMER 1.0

A newer version of DECIMER [45] evolves around the concept of transformers that have been successful for various tasks, such as NLP and Computer Vision (CV) problems. The update helped increase the accuracy of SMILES predictions from 90% to 96%, which is a significant development. The authors utilized a publicly available PubChem dataset to generate 2D molecular bitmap images of size $299 \times 299$ using CDK Structure Diagram Generator. The bitmap images were augmented using gaussian blur, salt and pepper noise, sharpening, brightness enhancement, and other methods. Apart from this, other two versions of the dataset were used as well, which included non-augmented images with and without stereochemical information. The model structure included the following steps:

1. Images were fed into pre-trained CNNs, such as InceptionV3 and EfficientNet-B3. The latter helped achieve better performance and was therefore used throughout the study. This step was performing a function of a feature extraction mechanism.
2. Unlike the previous version, the new DECIMER used SELFIES string instead of DeepSMILES. Tokenization was performed, after which images and their respective tokenized labels were converted into TFRecords, which is a data format used in TensorFlow that allows efficient training on Tensor Processing Unit (TPU).
3. The data is then fed into an encoder–decoder network with four encoder-decoder layers and eight attention heads. The authors have taken care of potential overfitting issues by adding a dropout of 0.1 to the network.
4. The model was trained on TPU. The training of the largest model took around 14 days, which is a significant improvement in the speed of training as compared to solutions before.

The advantages of the new transformer-based approach included the faster speed of training and better test performance. A maximum of 85.38% Tanimoto 1.0 similarity score has been achieved in the study. The system is completely open source, which provides open access to the results and serves as an important point of reference for future work.

### Models provided as solutions to Bristol Myers Squibb (BMS) contest on Kaggle

More recently, the BMS pharmaceutical company launched a competition on Kaggle to translate low-quality chemical structure images.

#### BMS dataset

The BMS dataset is a set of approximately 4 million synthetic molecular structure images generated and shared on Kaggle by the the aforementioned company. The images are arranged in a 3-level folder structure grouped by the image ID’s prefix. Training images are labeled with the corresponding InChI in a separate file.

The InChI labels have the following characteristics:

- Every label starts with ”InChI = 1S/”, which means that it is a standard InChI of version 1.
- Layers and sub-layers are separated with the “/” sign and prefix letters.
- There are six layers in total:
  - the main layer, which also contains the chemical formula sub-layer that is prevalent across all InChI labels, the atom connections and hydrogen sub-layers;
  - the charge layer;
  - the stereo-chemical layer;
  - the isotopic layer;
  - the fixed-H layer;
  - the reconnected layer.
- The maximum length of the label can be up to 200 symbols.

**Fig. 4:**
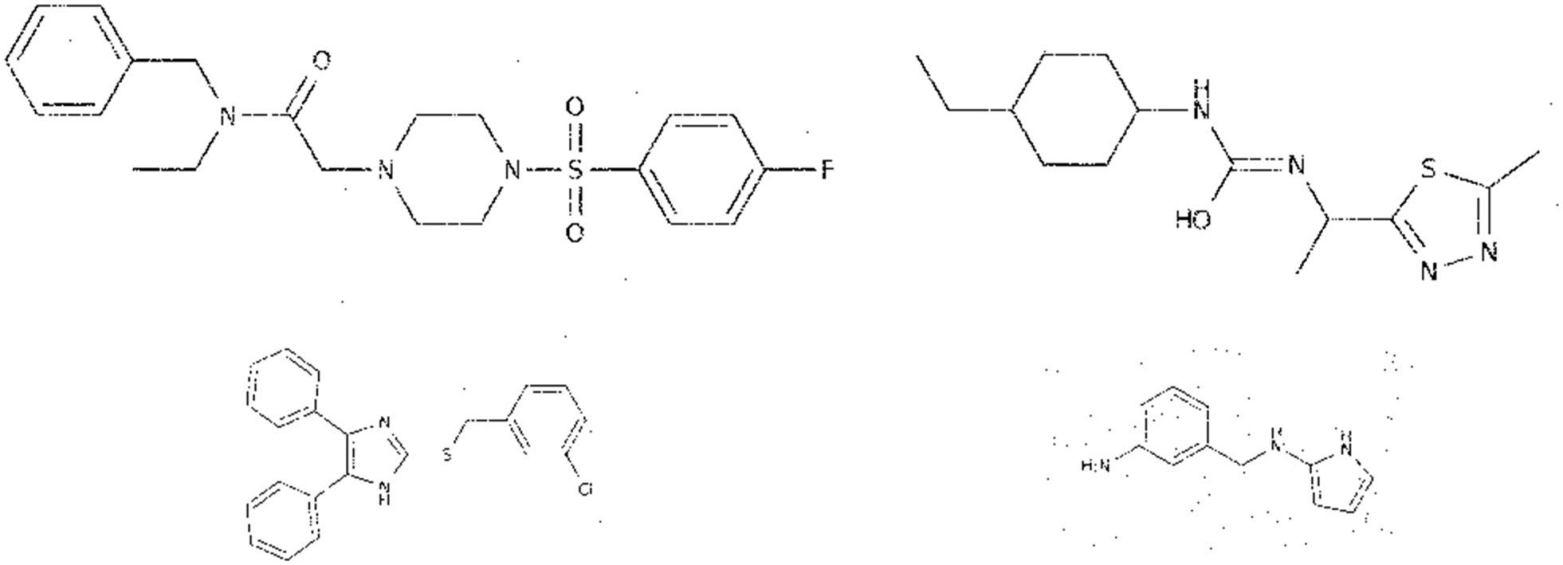
Samples from the BMS dataset: top row—training, bottom row—test samples

The images in the test subset differ from the train samples by their low resolution and quality (Fig. 4):

- they are often blurry,
- have more discontinued and/or removed image regions,
- contain salt and pepper noise, and
- unlike train images, which are usually horizontally positioned, test images can be vertically flipped or rotated by 90/270 degrees, such that the letters on the images change their orientation.

About 1.4% of train images and 0.7% of test images have an aspect ratio of more than 3:1. For example, such unusual image sizes as $1955 \times 72$ and $3043 \times 109$ are present amongst them. The maximum size of images in the train dataset is $1723 \times 1537$, whilst the minimum size is $117 \times 98$. In the test dataset, the maximum size observed is 1838x1578 and the minimum is $93 \times 123$.

**Fig. 5:**
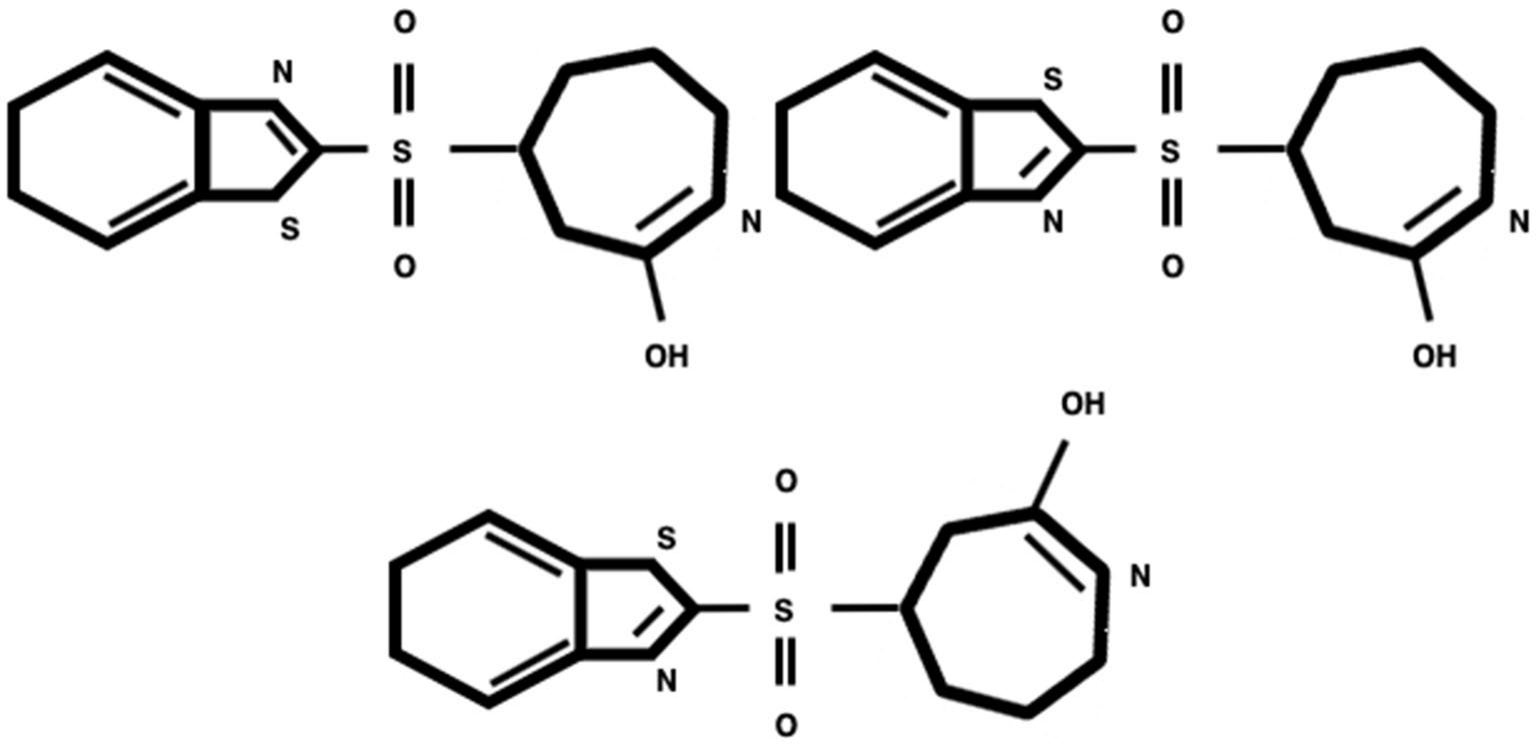
Three representations of the same formula: InChI=1S/C13H14N2O3S2/c16-12-8-9(4-3-7-14-12)20(17,18)13-15-10-5-1-2-6-11(10)19-13/h1-2,5-6,9H,3-4,7-8H2,(H,14,16)

Considering the mentioned shortcomings and variations, building a universal model becomes a challenging task. Additionally, the possibility of representing the same molecule with the variety of options requires models that consider the positional independence of the image elements from the label. Figure 5 displays an example of depicting the same molecule in different ways.

#### Evaluation metric

The measurement criterion for this problem is the Levenshtein distance (LD), which quantifies the difference between two strings. The higher the distance, the more dissimilar the strings. Mathematically, it is equal to the minimum number of edits required to equate the strings:

- if the last characters of strings *a* and *b* are the same, then LD is equal to the number of edits required up until those last characters;
- if the last characters of strings a and b are different, then LD is equal to the number of insertions, deletions and replacements needed to make string *a* equal to string *b*.

**Fig. 6:**
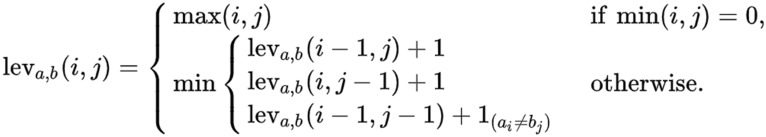
Levenshtein distance formula

The formula for the Levenshtein distance calculation is provided in Fig. 6.

As an evaluation criteria Levenshtein distance is a good option since it only considers the difference in parts, not only positional difference. For instance, there is one difference between the following two InChI sub-formula (2 versus 20), which would be evaluated as 58 by the Hamming distance (a positional difference— all the symbols do not match by position, including the last one):

c1-11- $$\underline{20}$$-21-16-10-19-17(12-5-3-2-4-6-12)14-9- 13(18)7-8-15(14)22

c1-11- $$\underline{2}$$-21-16-10-19-17(12-5-3-2-4-6-12)14-9- 13(18)7-8-15(14)22

### On sequence generation and InChI parsing methodology

Some of the BMS Kaggle solutions which have achieved a satisfactory result opted for an image-to-sequence modeling to approach the problem. This is an image captioning task that belongs to the CV and NLP domains, and is largely based on a CNN-based encoder and RNN-based decoder architecture. The encoder performs feature extraction from the images, which then serves as an input to the decoder to generate captions. The decoder generates the caption one character at a time, using the image features and previously predicted characters as inputs to predict the next one.

To make the model “understand” the input sequence, InChI strings must be parsed and encoded. Usually, a tokenizer-type class parses the InChI string in such a way that maps each chemical character to a unique integer index. The parser works in the following manner:

- Each InChI string is pre-processed in such a way that firstly, its chemical formula part (e.g., ‘C13H20OS’) is split into a separate set of atoms and their indices. For example, ‘C13’ becomes ‘C 13’.
- Secondly, its carbon part (part of the string between ‘/c’ and ‘/h’ characters, e.g. ‘/c1-9(2)8-15-13-6-5-10(3)7-12(13)11(4)14’) is split into separate characters by, firstly, parsing the zeroth symbol (the ‘/c’ sign) to preserve the slash sign in front of the carbon symbol. Then, the list of numbers following the carbon sign is parsed. Each individual number is dissected such that any surrounding symbols (e.g., ‘-’, ‘(’,‘)’ signs) are completely separated and the number kept “pure”. For instance, ‘/c1-9(2)8-15’ becomes ‘/c 1-9(2)8-15’.
- The processed InChI string then gets split into characters with the space delimiter. The unique parsed characters are added to a vocabulary.
- The vocabulary is sorted alphabetically in ascending order.
- Extra characters for “start”, “end” and “pad” of the string are also added as vocabulary members.
- Each vocabulary character is assigned a number, in ascending order. A string-to-integer dictionary is created.
- The reverse dictionary, i.e. integer-to-string mapping, is also maintained for backtracking purposes.
- Any text is then converted to a sequence of characters by first appending the “start” character to the beginning of the sequence, and then splitting the text into characters based on the space delimiter. Each individual character’s corresponding integer index gets added to the sequence list. Finally, the “end” character gets added to the end of the sequence list.
- To maintain backward compatibility, a sequence-to-text method is also maintained.

Some examples of relatively effective Kaggle solutions include:

- Long Short-Term Memory (LSTM) modeling with Attention network.
- Ensembles of Tree Network Trees, Vision Transformers and Attention.
- Ensembles of Vision and Vanilla Transformers as encoder and decoder, respectively, coupled with Swin encoder and Vanilla Transformer Decoder.
- CNN-based Encoder (e.g., Efficient Nets, ResNets) and an RNN-based Decoder with optional Attention network.
- Applications of beam search to identify the best output sequence.
- InChI sequence validation.

However, almost all of the cases have issues with accurate image augmentations, complex graph patterns, noise removals and image resolutions. The high-level design of the mentioned models is described below.

#### LSTM-based captioning

The task of an image captioning model is to generate a clear and correct description of a given image. Typically, an RNN based encoder-decoder structure is used for sequence-to-sequence translation tasks, whereby the encoder processes an input sequence, encodes it into a context vector, which then becomes the decoder’s initial hidden state. The decoder is responsible for generating the target sequence word at a time. The input to an image-captioning model is a multi-dimensional pixel array, and the output is its descriptive sequence. RNNs are typically used to map image vectors to sequences. There are two options of feeding images to RNNs: either flatten the image or generate its dense vector representation. The first approach works practically but results in a sparse matrix which is computationally inefficient to work with. Hence, CNNs are commonly used to extract image features.

CNNs implement transformations to the original input at different convolutional and pooling layers, thus creating useful feature maps which can serve as inputs to RNNs in place of flattened raw data. The extracted feature vector has a fixed length. Typically, CNNs are already pre-trained on a large dataset which significantly decreases the training effort. This is a transfer learning technique whereby a model trained on a classification task is reused for a different but related problem, saving compute time and resources.

**Fig. 7:**
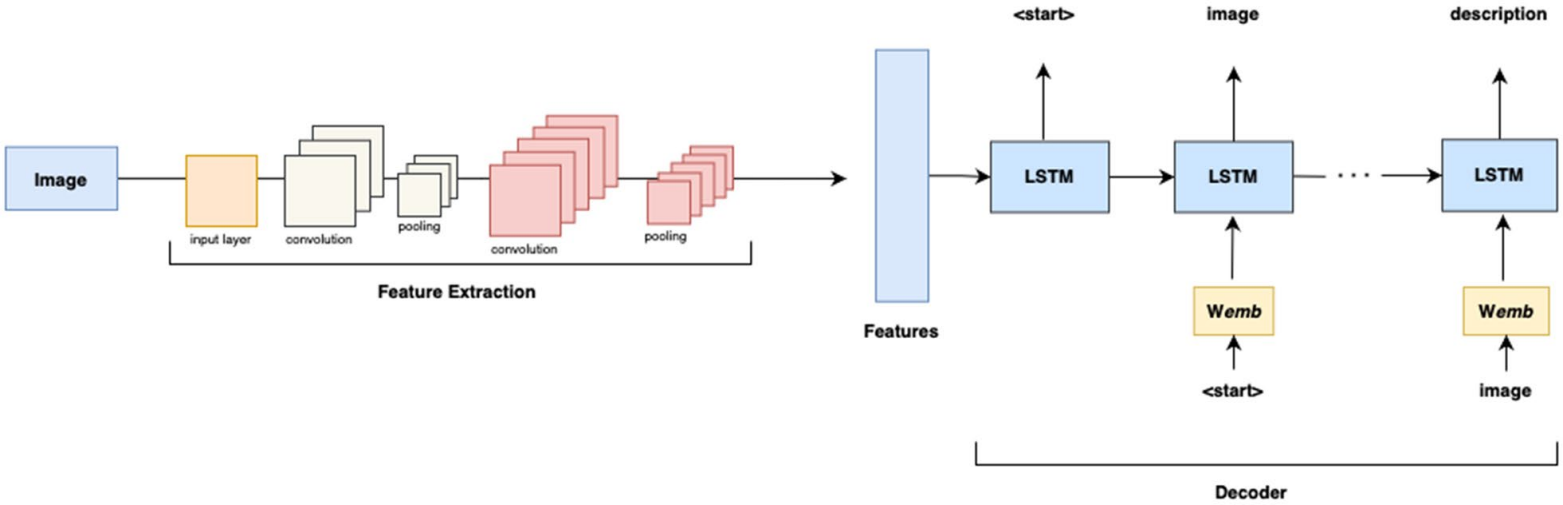
LSTM captioning

Regular RNNs are bad at capturing long-range dependencies due to the vanishing gradient problem: as the network grows in size, gradients become smaller in lower layers. Hence, to circumvent this problem, LSTM networks are utilized in practice as decoders. They are better at capturing long-range dependencies due to the existence of memory cells and gates in their internal structure: the memory cells remember previous states, while the gates control the flow of data from one state to the next. These help the network to carry on only the relevant information and omit the unnecessary information. Fig. 7 demonstrates the architecture of a basic LSTM-captioning model.

Despite the advantages over simple RNNs, LSTMs can also be forgetful. The matter lies in the requirement to compress all the important information from the source sentence into a fixed length context vector, which leads to a loss of necessary data (especially when it comes to longer sentences). In 2015, Bahdanau et al. [46] proposed a new methodology to focus on the important data in the source sequence by placing a relative importance score to each word in the context vector. This is the core of the attention mechanism [47] which is described in more detail in the following subsection.

The decoding algorithm outputs the probability of each word in the vocabulary being the next one in the generated sequence. Prediction stops as soon as the “stop” token or maximum string length is reached. There are two approaches to the model deciding which token to output next:

- *Greedy search*. This method selects the word with the highest probability at each position as the next prediction.
- *Beam search*. Instead of sampling once at each step, multiple word sequences are selected and kept as candidate sequences at every time step. The number of candidates is predefined by the *k* parameter which is the beam [48]. The final outputted sequence is the one with the highest total log probability over all generated characters. This is better than the greedy approach because it prevents the model from being stuck due to a bad decision at some stage of sequence prediction.

#### Attention model

In simple terms, the soft attention mechanism [6] works in the following way:

- The encoder outputs a matrix consisting of each hidden state, instead of a context vector.
- This matrix is fed into the attention model to compute the attention scores for each input word. The scores are used as weights to apply to the matrix.
- The weighted matrix is fed into the decoder, which allows the latter to focus on just the important bits of the input.

There is one problem with continuing to use RNNs as before, though - they work in a loop, processing one word at a time which creates a bottleneck in training. Hence, a novel stack-based encoder-decoder structure (“Transformer”) is suggested:

- An encoder stack consists of several individual encoders, feeding into one another sequentially. The input to the first encoder is an embedding vector of the input sequence concatenated with positional encoding of words in the sentence. Encoders are in fact CNNs, which usually utilize transfer learning techniques, i.e. they are pretrained (for example, ResNets, pretrained on ImageNet data). However, since encoding is performed instead of classification, the last pooling and linear layers are dropped. An adaptive average pooling layer is added instead to make sure that all encodings are of the same size, regardless of the original image size.
- A decoder stack consists of several individual decoders that receive inputs from each other and from the final encoder state. The input to the first decoder is an embedding vector of the target sequence concatenated with positional encoding of words in the sentence.
- Within each encoder, there is a self-attention model, whereby the input sequence pays attention to itself.
- Within each decoder, a self-attention layer is also present, whereby the target sequence pays attention to itself.
- An encoder-decoder attention layer in the decoder allows the target sequence to pay attention to the input sequence.
- The attention layer accepts three inputs— Query, Key and Value to compute the attention scores for each word according to this formula: $score = {softmax(\frac{(QK^{T} + Mask)}{\sqrt{embedding size}}) * V}$,

here Q is Query (word for which attention score is computed), K is Key and V is Value (words to which attention is paid). The dot product between Q and K defines the similarity of the words. The calculated scores are indicative of the probability that a particular word in the vocabulary occurs at a certain position in the sentence. For example, if the length of the target sequence is 3 words and the target vocabulary has a total of 1000 words, then 1000 scores are generated per each of the 3 words. Softmax activation is then applied to return the computed scores as likelihoods.

**Fig. 8:**
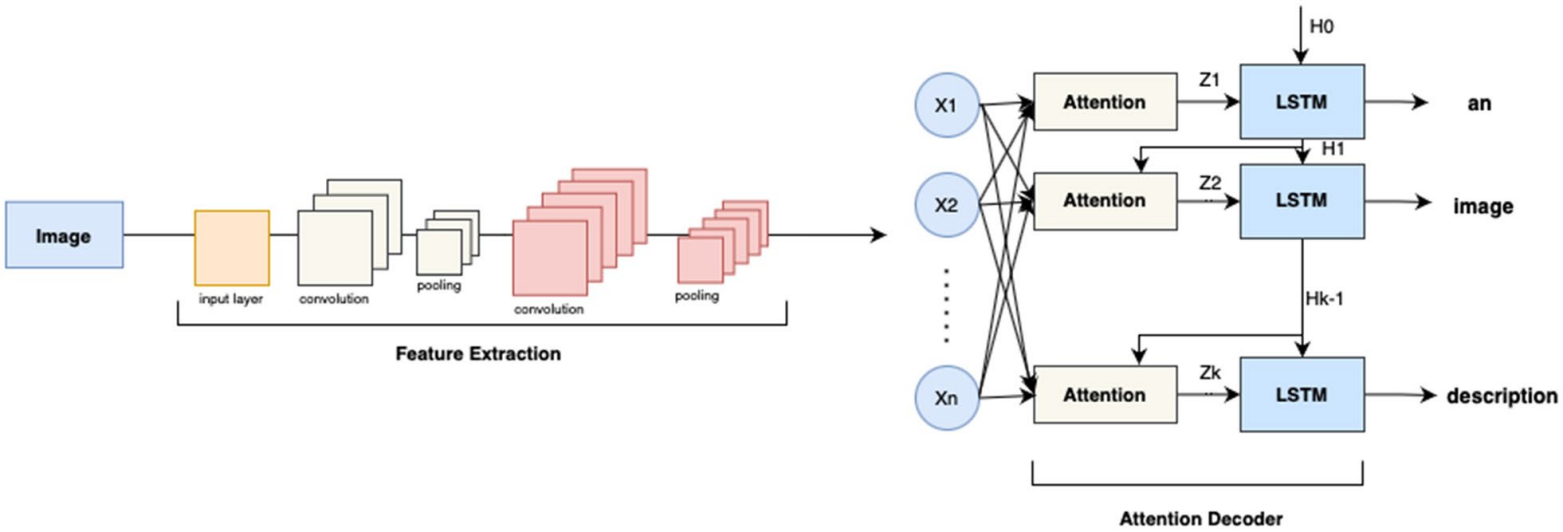
LSTM captioning with attention

Figure 8 depicts the basic framework of the LSTM with the Soft Attention image captioning model described above.

#### Vision transformers

**Fig. 9:**
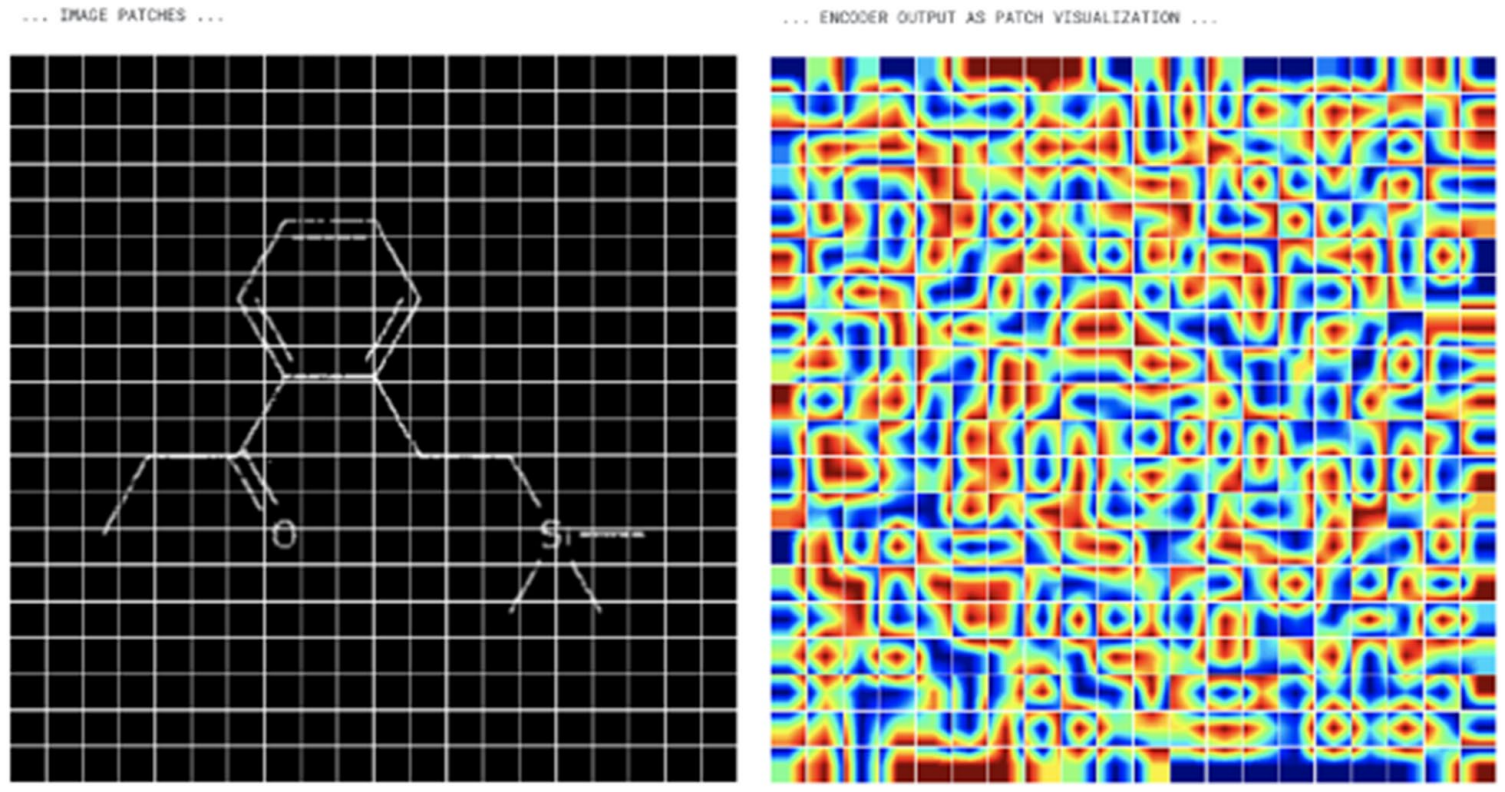
ViT encoder input and output

The use of Vision Transformers (ViT) for the problem of OCSR is potentially due to the possibility of using Transformer architecture for computer vision tasks [7]. The resulting architecture is a combination of Vision Transformer encoder and Transformer decoder. The details of implementation are the following:

- The input image is resized to a common shape.
- The image is divided into 2D patches of $16 \times 16$ pixels. This configuration can be overridden and a different patch size may be chosen. Example image divided into patches is shown in Fig. 9 (left).
- Patches are flattened and positional encoding is added.
- The data is passed through a ViT encoder. Instead of passing the item to a Multi-Layer Perceptron (MLP), as in the original paper, the data is passed to a Transformer decoder. The output of this step is shown in Fig. 9 (right).
- Transformer decoder receives output embedding and encoder output as its input and processes the information.
- The output of the Transformer decoder is used to generate a sequence of InChI label.

This approach allows usage of Transformers for the task of OCSR, and can be used for solving the problem. However a huge amount of data is needed to train such a model. Additionally, the number of epochs should also be high. This leads to the point that the task is resource intensive and requires extensive computational power.

#### Image captioning model based on deep transformer-in-transformer (ICMDT)

This paper is the most recent development focusing on the problem as an image captioning task, whereby an encoder-decoder structure with an optional attention model is often used [49]. The analysis is conducted on the Bristol-Myers Squibb Kaggle dataset of chemical structures and the authors managed to achieve a Levenshtein distance score in the range of 0.24–2.5 by improving the standard Transformer-in-Transformer (TNT) block. The suggested model outperforms its peers both in terms of accuracy results and speed of convergence.

On a granular level, the problem is to automatically generate InChI equivalents of images containing chemical structures. Hence, the problem is the fusion of computer vision and natural language processing fields.

From the standpoint of image pre-processing, the researchers apply the following:

- image reshaping into a square form and subsequent filling of insufficient parts with mid-image pixels;
- adding noise, blur and random 90 degree rotations to the train dataset to better simulate test dataset quality;
- image denoising;
- smart cropping;
- padding the image to keep a consistent aspect ratio.

**Fig. 10:**
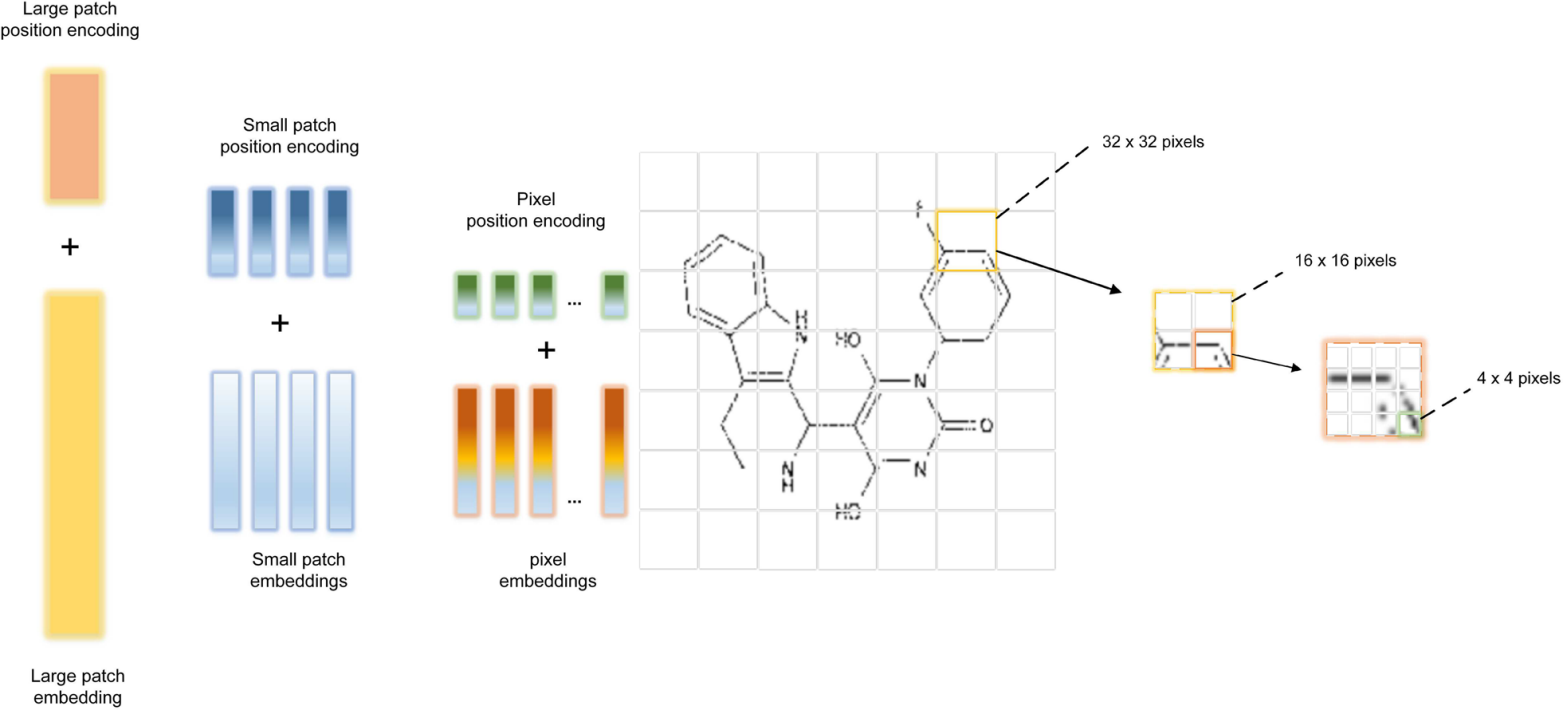
Division into patches and positional encoding [49]

The authors achieve an outbreak due to improving the regular models used by previous researchers. Specifically,

- The TNT block is deepened into three separate parts:

1. an exterior transformer block, which processes large patch embeddings (these are sequences of small patch embeddings);
2. a middle transformer block, which processes small patch embeddings (these are sequences of pixel-level features);
3. an internal transformer block, which processes the pixel-level features contained in small patch embeddings.

- An image is divided into *n* non-overlapping 32x32 patches (the large patches), which then unravel into 16x16 smaller patch embeddings, and finally 4x4 pixel-level tensors. The unraveling takes place by passing the embeddings through linear layers. The division process is illustrated in Fig. 10 (top).
- Position encoding is added to each small patch and pixel embedding (Fig. 10 (bottom)).
- Training is performed by first, using images at a resolution of $224 \times 224$ and then changing the resolution of those images that have label lengths exceeding 150 to $384 \times 384$ for fine-tuning (the assumption is that longer labels correspond to more complex structures). Label smoothing technique is applied to regularize the noise in target strings, and noisy labels are applied in general to continue predicting the string despite the incorrect prediction of the previous character in the sequence. Optimizers in use are Lookahead and Rectified Adam (RAdam), and the loss function is anti-focal.
- Test dataset is also rotated by ninety degrees in any direction. The tested batch size ranges from 16 to 64+ and the size is kept constant once the validation loss becomes stable.
- Beam search with k set to 16 is used in the inference stage.

The results of the novel approach outperform comparison models (including Kaggle model ensembles) at circa seventh epoch and convergence takes place at approximately tenth epoch. The authors find that denoising both train and test images improves the outcome (or, vice versa, i.e. adding noise to train set so that it is more aligned with the test images), while padding has no impact and smart cropping diminishes the performance.

However, the model fails to determine some stereo-chemical layers completely and is error-prone around “+/−” signs. Also, it is difficult to say how the methodology will work on images outside the Kaggle dataset, such as PubChem, which can contain more complex and noisier images.

**Table 2:** Performance of the ML-based approaches

OCSR system	Year	Dataset	Classification	Details	Evaluation	Results
CNN based approach from [40]	2019	3600 images created by 200 people (90/10 for training and test)	Ring structures	Transfer learning with VGG-19 for 36 classes	Recognition rate	80%
MSE-DUDL	2019	Training: more than 50 million samples from PubChem, Indigo and USPTO datasets Test: 454 (Valko dataset) and other proprietary datasets	SMILES	U-Net based segmentation with GridLSTM	Not given	Validation: 77%-82% Test: 41% (Valko), 83%(others)
DECIMER	2020	PubChem	DeepSMILES	Encoder/Decoder model with CNN and GRU	Tanimoto	0.53
DECIMER Segmentation	2021	Training: 994 articles from the Journal of Natural Products Test: 777 pages from 75 journals (Journal of Natural Products, Phytochemistry and Molecules)	segmented structures	Mask R-CNN for the object detection and VGG for classification	Recognition rate	91.3%
DECIMER 1.0	2021	39 million (PubChem) (90/10 for training and test)	SELFIES	Transfer learning (EfficientNet) for classification and transformer for sequence generation	Tanimoto	0.99 96.47% of the results had Tanimoto = 1.0
ICMDT	2021	4 million images (BMS Dataset): 2.4 million training 1.6 million test	InChI	Deep TNT block	Levenshtein distance	2.5

A brief description of the mentioned ML-based approaches is depicted in Table 2. Compared to rule-based approaches, these systems use much bigger set of the training datasets and deliver higher results. There is still disunity in the measurement of the results: some of the approaches measure the results by standard ML criteria—recognition rate, accuracy and precision—others use distance measurements for the string. These and the other points are discussed in the Conclusions section.

#### Gaps of ML-based systems

1. Super-atoms and complex elements are usually underrepresented and not well classified in most existing solutions.
2. Image resolutions are not well handled: some models work well with low-resolution images, while others with high-resolution. There is no unified solution.
3. Datasets used were not large enough to achieve the highest possible performance.
4. DECIMER Segmentation mainly works well with raster images. There is a need for a similar system for new vector images as well.

## Conclusion

The evolution of the OCSR systems starts with rule-based expert systems with less computationally complex processes and ends with complex models that require almost no guidance towards the problem and consume huge computational resources. Despite the exceptional difference between the latter, the rule-based approach and heuristics shall not be neglected completely - authors believe that a good combination of the two may deliver better results. The further recommendations are correspondingly categorized for both approaches:

### Machine learning models

As mentioned at the beginning of the paper, the problem belongs to the image captioning problem and the best results are achieved with the latest models that involve attention-based image decoders and NLP-based text decoders. The realization and results follow the trend of the model evolution: Transfer learning+RNNs with Attention, Transfer Learning+Transformer, ViT Transformer, and involvement of more powerful NLP models. The authors recommend the consideration of the following items during the ML-based model design:

1. The efficiency of the utilized Transfer learning model and methodology shall be analyzed. The analysis shall cover the statistical evaluation of the feature distribution for the same, similar, and different chemical structures.
2. Chemical formula images are usually provided as binary images, as shown in black drawings on a white background. This encodes the background and chemical structure pixels as positive (1) and negative (0) signals, respectively. Feeding the network with such a setup may deliver relatively poor results since the model will be trained for the distinction of the backgrounds. The inversion of the image as it is practiced in the majority of the latest systems may increase the quality of recognition.
3. Analysis of the image quality in the dataset is important. If the data has too much noise and discontinuities in shapes, it would be a good idea not to crop the images before the resizing. The empty pixels that stand for the removed strokes on the surroundings may deliver additional information to the model.
4. Variations of the image size and ratio shall be analyzed for the proper cropping and resizing strategy: some of the chemical structures may be depicted in an unusual image ratio (there are plenty of images in public databases that exceed the 1:5 ratio).
5. The bigger dataset helps the model better “understand” the problem. In such a case, extra augmentation and increasing the model complexity may prolong the training time, which may take days or weeks of running on GPU.
6. Levenshtein Distance seems a good evaluation criterion but the average value of it may not deliver the right information on the model’s performance. Consideration of the other string sequence similarity algorithms (customized for the chemical structure description) might be useful. Variations in one symbol may present with different severity for different cases: confusion of the chemical elements (“F” instead of “S”) might be much more serious than confusing numbers. Customization of Optimal Matching (OM) metrics for the particular sequence description type can be developed to address the specific problem. Alternatively, the Tanimoto coefficient can be applied, which measures the ratio of the intersection of the two sets over the union of the two sets. [50, 51] provide analysis of the distance and similarity metrics used in sequences comparison. [52] introduces a distance measure based on q-grams, and describe its implementation for the circular sequence comparison.

### Rules and heuristics

1. Since the chemical structure drawings are at an invariant position invariant, the corresponding augmentation methods might be used. The images either can be rotated and flipped by saving the text orientation or variations of the structures can be generated based on the given formula.
2. The sequence of the decoder shall be paired with a statistical method like Beam Search. The latest models use the Beam Search algorithm with a depth between 15 and 20.
3. The generated formula shall be validated and corrected according to the rules: the order, sequence, the elements (if any element is detected in the general part but not mentioned or was misspelled in the connection part), numbers, and the formula structure shall be checked for the correspondence with the standard and fixed with the best effort. The formula validation on average increases the accuracy of systems by around 5–6%.
4. For the efficiency of the ML part, the chemical structures can be classified before the training and inference. Various image analysis methods, rules (image analysis) or ML-based clustering can be used for the division of the structures into 3–5 kinds and can train them by different models, which may use a different approach that fits each.
5. Since CNNs are sensitive to image rotation, affine transformation, and are unable to capture the spatial relation among the parts, using Capsule Networks may deliver better results [8–10]. Capsule Networks replace scalar-output feature detectors with vector-output capsules and max-pooling with routing-by-agreement which adds position invariance. With such an approach, the capsule neurons represent the existence of the object in an image and its various features: orientation, position, size, velocity, texture and deformation.

Lack of a standardized dataset challenges OCSR development more than mentioned technical difficulties. Unlike image classification, object detection and segmentation datasets [53–56], there is no standard chemical structure dataset that could be used for the comparison of the results of various approaches. Such a dataset needs to contain images of the agreed quality and size (some of the samples in contest datasets were not readable at all), provide labels at least in SMILES and InChI and define exact measurement criteria. Differentiation of the image or formula complexity (2D/3D, molecule type, string length or number of nested elements) would add fairness in the comparison of various models. Considering the complexity of the initiative, authors see the realization of this project by the open contribution of specialists, moderated by the researchers of the leading institutions.

## Data Availability

The dataset used to support conclusions in [38] is available on GitHub ChemType 2 repository, https://github.com/bradleyemi/chemtype2 [57]. The dataset used to support conclusions in [40] is available on GitHub Offline Handwritten Chemical Notations Datasets repository, https://github.com/Septemberxin/Offline-Handwritten-Chemical-Notations-Datasets [58].

## Abbreviations

ASCII: American Standard Code for Information Interchange
BMS: Bristol Myers Squibb
CAS RN: Chemical Abstract Service Registry Number
CDK: Chemistry Development Kit
CLiDE: Chemical literature data extraction
CNN: Convolutional neural network
CV: Computer vision
DECIMER: Deep learning for chemical image recognition
DNN: Deep neural networks
GPU: Graphics processing unit
GRU: Gated recurrent unit
ICMDT: Image captioning model based on deep transformer-in-transformer
InChI: International chemical identifier
OCR: Optical character recognition
OCSR: Optical chemical structure recognition
OROCS: Optical recognition of chemical graphics
OSRA: Optical structure recognition application
LD: Levenshtein distance
LSTM: Long short-term memory
ML: Machine learning
MLP: Multi-layer perceptron
NLP: Natural language processing
OM: Optimal matching
ReLU: Rectified linear unit
RNN: Recurrent neural network
SMARTS: SMILES arbitrary target specification
SELFIES: SELF-referencIng embedded strings
SMILES: Simplified molecular-input line-entry system
SVM: Support-vector machine
TNT: Transformer-in-transformer
TPU: Tensor processing unit
UNII: Unique ingredient identifier
USPTO: United States Patent and Trademark Office
VGG: Visual geometry group
ViT: Vision transformer
